# Bladder Irritation without Mesh Penetration after Hernia Repair: A Case Report

**DOI:** 10.70352/scrj.cr.25-0472

**Published:** 2025-10-15

**Authors:** Kenichi Ishibayashi, Maika Zasu, Yusuke Ikku, Tetsuya Asakawa, Katsuya Gunjigake, Takahisa Yamaguchi, Yoshinao Ohbatake, Shiro Terai, Hirotaka Kitamura, Shinichi Kadoya

**Affiliations:** Department of Gastroenterological Surgery, Ishikawa Prefectural Central Hospital, Kanazawa, Ishikawa, Japan

**Keywords:** hernia, bladder irritation, mesh, postoperative complication

## Abstract

**INTRODUCTION:**

Bladder irritation after inguinal hernia repair is typically associated with mesh migration into the bladder. Notably, no previous cases have described bladder irritation symptoms in the absence of direct mesh penetration. This report presents a rare instance of bladder symptoms caused by a folded mesh, despite normal cystoscopic and cystographic findings.

**CASE PRESENTATION:**

A 49-year-old woman presented with urinary urgency, incontinence, and chronic right inguinal pain for 3 years, 8 years after undergoing inguinal hernia repair using the modified Kugel method. Although cystoscopy and cystography revealed no abnormalities, pelvic CT and MRI demonstrated a curved fatty tissue protruding toward the bladder. Laparoscopic exploration confirmed the presence of a folded mesh adjacent to the bladder wall. The mesh was successfully removed and a new mesh was placed, resulting in complete resolution of the urinary symptoms and pain without postoperative complications.

**CONCLUSIONS:**

In patients presenting with bladder symptoms after inguinal hernia repair, mechanical irritation of the bladder wall by mesh should be considered—even when cystoscopic and radiographic findings are normal.

## INTRODUCTION

Although complications of inguinal hernia repair are relatively uncommon, they can significantly impact a patient’s quality of life when they arise. Among these, mesh migration or intrusion into adjacent organs—particularly the urinary bladder—is considered a rare but clinically significant complication. In prior reports of mesh penetration into the bladder, common symptoms included increased urinary frequency (pollakiuria), painful urination (dysuria), recurrent urinary tract infections, and hematuria. These symptoms are generally attributed to mechanical irritation or direct penetration of the bladder wall by the mesh.^1,2)^ However, to date, no cases have described bladder irritation symptoms in the absence of direct mesh penetration into the bladder.

Here, we describe a rare case of bladder irritation after inguinal hernia repair using the modified Kugel method, which is sometimes referred to domestically in Japan as the “direct Kugel” method, in the absence of direct mesh penetration into the bladder. The patient’s symptoms significantly improved after laparoscopic mesh removal.

## CASE PRESENTATION

A 49-year-old woman presented with urinary urgency, urinary incontinence, and right-sided inguinal pain. She had previously undergone right inguinal hernia repair via the modified Kugel method 8 years earlier. Three years prior, she began experiencing urinary urgency, incontinence, and persistent right-sided inguinal pain. She initially received conservative treatment with analgesics at a local clinic, but her symptoms persisted, and she was referred to our hospital for further evaluation and treatment. The patient’s height, weight, and body mass index were 161.0 cm, 43.5 kg, and 16.8 kg/m^2^, respectively. A surgical scar was observed in the right inguinal region, and a firm mass was palpable at the site. The patient reported chronic pain at the site, with a numerical rating scale score of 3/10 and localized tenderness. Routine blood tests and urinalysis revealed no abnormalities.

**Fig. 1:**
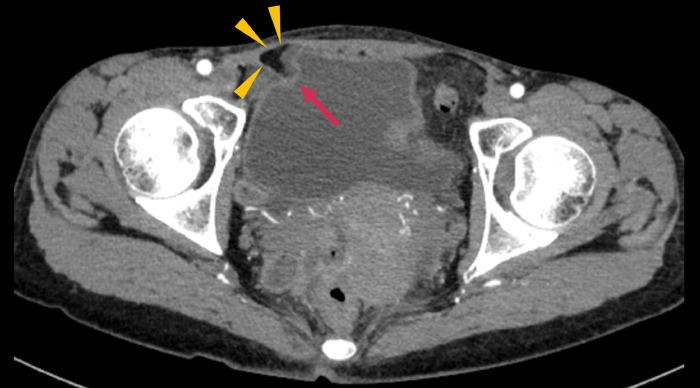
Findings from CT. CT revealed curved fatty tissue (arrowheads) protruding toward the bladder (arrow) from the right inguinal region.

CT revealed curved fatty tissue protruding toward the bladder from the right inguinal region; however, the mesh itself could not be visualized (**Fig. 1**). MRI revealed a corresponding curved low-signal structure on T2-weighted sequences. Urinalysis revealed no abnormalities, including negative hematuria. Urine culture and cytology were not performed. Cystoscopy demonstrated no abnormal findings, with no evidence of tumor, bladder wall deformity, or mucosal changes. Cystography showed neither contrast medium leakage nor bladder deformation.

**Fig. 2:**
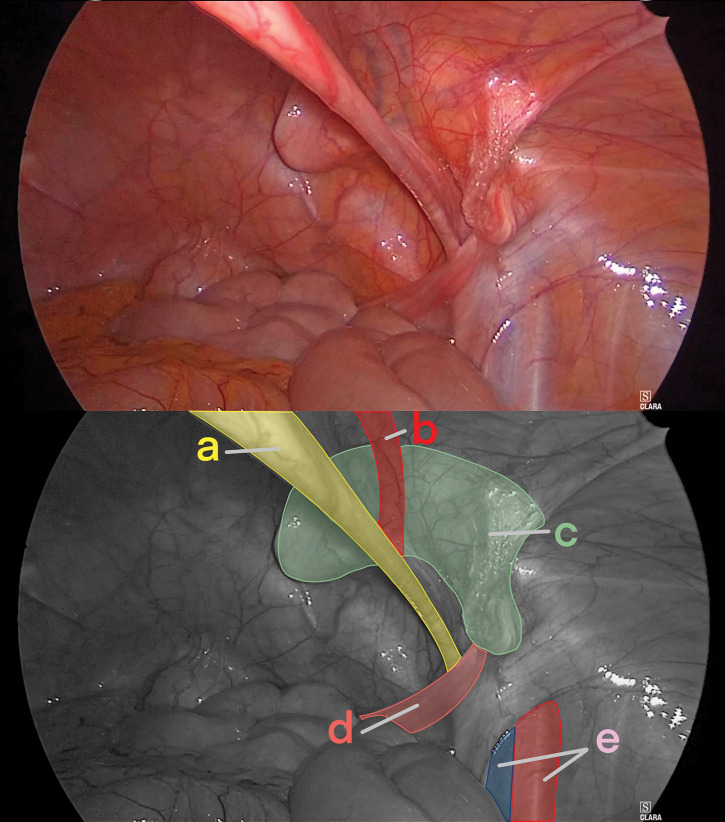
Intraoperative findings. A curved mesh protruding into the right pelvic cavity is seen. (a) Medial umbilical fold, (b) inferior epigastric vessels, (c) curved mesh, (d) round ligament of the uterus, and (e) femoral vessels.

Given the diagnosis of bladder irritation and chronic post-hernia repair pain secondary to mesh curvature, laparoscopic mesh removal was planned. The operation was initiated using 3 ports. Upon inspection of the abdominal cavity, a curved mesh was identified in the right inguinal region (**Fig. 2**). After incising the peritoneum and dissecting the preperitoneal and prevesical spaces, dissection was carried out along the mesh. The mesh was easily dissected from the bladder. As dissection between the mesh and the inferior epigastric vessels proved difficult, the vessels were transected both proximally and distally. No evidence of nerve entrapment within the mesh was observed. The mesh was removed, and a new mesh (3DMax Light Mesh, Large size, Bard; BD, Franklin Lakes, NJ, USA) was implanted and fixed with the SorbaFix absorbable fixation system (BD). The peritoneum was reapproximated, and the operation was concluded. The operative time was 207 min, and the estimated blood loss was 1 mL.

The patient was discharged on POD 2 without complications. At the 2-week follow-up, spontaneous pain had resolved, mild tenderness persisted, and bladder irritation symptoms had fully resolved. At the 1-month follow-up, both spontaneous pain and tenderness had resolved, with no recurrence of bladder symptoms. At the 6-month follow-up visit, the patient remained free of pain and bladder symptoms, with no evidence of recurrence.

## DISCUSSION

This case indicates that bladder symptoms after inguinal hernia repair were resolved following mesh removal. Even when cystoscopy and cystography reveal no abnormalities, clinicians should consider the possibility that symptoms may be caused by a curved mesh. Intravesical mesh migration is typically the primary concern when bladder irritation symptoms occur after inguinal hernia repair. The most frequently reported symptoms include pollakiuria (38%), gross hematuria (34%), and recurrent urinary tract infections (31%). The median interval between the initial hernia repair and the diagnosis of mesh migration is 60 months (range: 3–300 months).^3)^ Cystoscopy and pelvic CT are useful for diagnosis, and treatment typically involves mesh removal, with partial bladder resection performed when necessary.^4–6)^

To our knowledge, no previous reports have described a case in which cystoscopy or cystography showed no abnormalities, yet CT and MRI identified a curved fatty mass adjacent to the bladder, suggesting mechanical stimulation caused by a curved mesh. One possible reason why such cases have not been previously reported is that the mesh is often not visualized on CT, making it difficult to diagnose a curved mesh as the cause of bladder irritation. Therefore, it is possible that some symptomatic patients remain undiagnosed and fail to receive appropriate treatment. Furthermore, this case may represent an extremely rare prodromal stage of intravesical mesh migration. In reported cases of mesh penetration into the bladder after hernia repair, cystoscopy was the key examination in 48% of cases and CT in 26%.^3)^ When the mesh penetrates the bladder, disruption of the bladder wall serves as the diagnostic clue. However, when the mesh does not penetrate the bladder and cystoscopy shows no abnormalities, diagnosis is more challenging because the mesh itself is usually not visible on CT.^7)^ The major factors that determine mesh visibility on CT are density, structure (woven or knitted), and thickness. Polypropylene- and polyester-based meshes are isodense to muscle and therefore not visualized unless there is a fat interface or metallic tackers.^8)^ In our case, curved fatty tissue was observed, leading us to suspect folding of the mesh and reach the diagnosis. We believe that, in patients with a history of inguinal hernia repair, the presence of curved fatty tissue in the inguinal region on CT should raise suspicion of a folded mesh.

The immediate resolution of symptoms after mesh removal strongly supports the hypothesis that mechanical stimulation from the mesh induced bladder wall hypersensitivity. Chronic mesh-induced stimulation may lead to nerve sensitization and localized inflammatory responses.^4,9)^ Even without direct invasion of the bladder wall, irritation from adjacent foreign material may impair bladder function.^10)^ In particular, the Kugel patch used in the modified Kugel method contains a memory recoil ring, and its shape and rigidity are considered to increase the contact pressure on surrounding tissues, potentially causing chronic pain and mechanical irritation of adjacent organs even without penetration.^11)^ The clinical course, characterized by symptom exacerbation during physical activity, further supports the role of mechanical contact or traction-induced stimulation. In previous reports, further evaluation of bladder irritation symptoms frequently resulted in a diagnosis of intravesical mesh migration. In such cases, bladder wall irritation, similar to that observed in our patient, may have represented a prodromal stage prior to actual penetration.^12)^

Chronic pain after hernia repair can be classified into nociceptive pain, neuropathic pain, or a mixed type, depending on the underlying mechanism.^13,14)^ Differentiation between these pain types relies heavily on physical and clinical findings. Nociceptive pain is characterized by a localized tender point with a clearly identified maximal point of pain, and sometimes a palpable hard nodule. Neuropathic pain, on the other hand, is characterized by tenderness along the dermatome of the affected nerve. There is no standardized treatment for chronic pain after hernia repair, and surgical approaches vary. Some reports recommend mesh removal alone, whereas others suggest neurectomy or a combination of neurectomy and mesh removal.^15)^ In our case, the patient’s symptoms were consistent with nociceptive pain, without findings suggestive of neuropathic involvement. Therefore, we determined that neurectomy was unnecessary and performed only mesh removal.

In our case, the laparoscopic approach proved effective for dissecting the mesh from the bladder. In previously reported cases of mesh penetration into the bladder, laparoscopic mesh removal—often accompanied by partial bladder resection when necessary—has been shown to be a useful strategy.^5,7)^ Even in cases without direct penetration, as in our patient, bladder irritation symptoms may indicate adhesion between the mesh and the bladder wall. Therefore, we believe that a laparoscopic approach is also preferable in such cases, as it allows for safe dissection of potential adhesions, similar to cases with confirmed mesh penetration.

## CONCLUSIONS

In patients presenting with bladder symptoms after inguinal hernia repair, mechanical irritation of the bladder wall by mesh should be considered—even when cystoscopic and radiographic findings are normal.
